# Chronic Kidney Disease Associated with Ischemic Heart Disease: To What Extent Do Biomarkers Help?

**DOI:** 10.3390/life14010034

**Published:** 2023-12-25

**Authors:** Maria-Ruxandra Cepoi, Stefania Teodora Duca, Adriana Chetran, Alexandru Dan Costache, Marilena Renata Spiridon, Irina Afrăsânie, Sabina Andreea Leancă, Bianca-Ana Dmour, Iulian Theodor Matei, Radu Stefan Miftode, Larisa Miftode, Cristian Sorin Prepeliuc, Mihai Ștefan Cristian Haba, Minerva Codruța Bădescu, Irina Iuliana Costache

**Affiliations:** 1 Department of Internal Medicine, Faculty of Medicine, “Grigore T. Popa” University of Medicine and Pharmacy, 700115 Iași, Romania; cepoi_maria-ruxandra@d.umfiasi.ro (M.-R.C.); stefania-teodora.duca@d.umfiasi.ro (S.T.D.); adriana-ion@email.umfiasi.ro (A.C.); demsa-irina@d.umfiasi.ro (I.A.); sabina-andreea.leanca@d.umfiasi.ro (S.A.L.); bianca-ana-dmour@email.umfiasi.ro (B.-A.D.); matei.theodor-iulian@d.umfiasi.ro (I.T.M.); radu-stefan-miftode@email.umfiasi.ro (R.S.M.); mihai.haba@umfiasi.ro (M.Ș.C.H.); minerva.badescu@umfiasi.ro (M.C.B.); irina.costache@umfiasi.ro (I.I.C.); 2 Department of Cardiology, “St. Spiridon” County Clinical Emergency Hospital, 700111 Iași, Romania; marilena.spiridon@umfiasi.ro; 3 Department of Cardiovascular Rehabilitation, Clinical Rehabilitation Hospital, 700661 Iași, Romania; 4 Department of III Internal Medicine Clinic, “St. Spiridon” County Clinical Emergency Hospital, 700111 Iași, Romania; 5 Department of Infectious Diseases, Faculty of Medicine, “Grigore T. Popa” University of Medicine and Pharmacy, 700115 Iași, Romania; ionela-larisa-miftode@email.umfiasi.ro (L.M.); cristian-sorin-cs-prepeliuc@email.umfiasi.ro (C.S.P.); 6 “St. Parascheva” Clinical Hospital of Infectious Diseases, 700116 Iași, Romania

**Keywords:** chronic kidney disease, coronary artery disease, biomarkers, inflammation, acute coronary syndrome

## Abstract

Chronic kidney disease represents a complex and multifaceted pathology characterized by the presence of structural or functional renal anomalies associated with a persistent reduction in renal function. As the disease progresses, complications arise due to the chronic inflammatory syndrome, hydro-electrolytic disorders, and toxicity secondary to the uremic environment. Cardiovascular complications are the leading cause of death for these patients. Ischemic cardiac pathology can be both a consequence and complication of chronic kidney disease, highlighting the need to identify specific cardiorenal dysfunction biomarkers targeting pathophysiological mechanisms common to both conditions. This identification is crucial for establishing accurate diagnoses, prognoses, and risk stratifications for patients. This work is intended to elucidate the intricate relationship between chronic kidney disease and ischemic heart disease and to investigate the roles of cardiorenal biomarkers, including cardiac troponin, natriuretic peptides, galectin-3, copeptin, fibroblast growth factor 23 and its co-receptor Klotho, soluble suppression of tumorigenicity 2, and plasma growth differentiation factor 15.

## 1. Introduction

Chronic kidney disease (CKD) represents a significant global health issue, affecting between 8% and 16% of the global population [1]. Over the past two decades, the prevalence of CKD has increased more rapidly compared to other chronic conditions [2]. Of the population affected by CKD, approximately 79% have end-stage kidney disease (ESKD) [3,4]. Accurately estimating prevalence is challenging due to the often-asymptomatic initial stages. The World Health Organization predicted that there are over 5–10 million CKD-related deaths annually, with diagnosed cases projected to surpass 4 million by 2036 [5]. Chronic kidney disease is a complex and multifaceted pathology defined by the presence of structural or functional renal anomalies associated with a persistent reduction in renal function lasting more than 3 months, progressing to ESKD and often accompanied by cardiovascular disease [3,6,7,8].

Patients with CKD are associated with multiple comorbidities that contribute to an additional risk of mortality and an increase in healthcare-associated costs. Cardiovascular complications, responsible for 50% of advanced CKD deaths, include coronary artery disease (CAD), heart failure, arrhythmias, and sudden cardiac death [1,8]. Ischemic heart disease is a pathological condition characterized by an imbalance between the supplied and demanded oxygen resulting from a reduction in cardiac blood flow, and the most common cause is coronary artery disease (CAD) [9]. CAD prevalence rises with declining GFR, leading to a twofold higher mortality risk at eGFR 30–59 mL/min per 1.73 m^2^ and a threefold higher risk at eGFR 15–29 mL/min per 1.73 m^2^ compared to those with normal renal function [7,10]. Currently, CKD associated with CAD poses a significant challenge in terms of making medical decisions given that the symptoms, cardiorenal biomarkers, and non-invasive studies relating to myocardial ischemia evaluation have varying thresholds of sensitivity and specificity within this patient group [7]. Another crucial aspect for this patient category is the uncertainty regarding the efficacy of interventional or pharmacological treatment, as most clinical studies exclude patients with advanced CKD. The consequence is the undertreatment of cardiovascular risk factors and inappropriately low rates of coronary angiography for patients with CKD, a phenomenon also known as “renalism” [7,11].

The relationship between the kidneys and the heart is bidirectional. The outcome of this connection involves maintaining fluid homeostasis, an acid–base balance, and blood pressure. Renal dysfunction leads to a disruption in the excretion of salts and water, resulting in increased cardiac preload and afterload, as well as the accumulation of toxic metabolites [7,12]. Additionally, a toxic environment is established, influenced by uremia and inflammation, leading to specific changes discussed in this paper. There are common mechanisms in both CKD and CAD, and they are newly described, necessitating the exploration of new treatment strategies that target novel therapeutic endpoints and identify common cardiorenal biomarkers with roles in prognosis diagnosis, in addition to therapeutic monitoring for these patients. In recent years, there have been several significant developments regarding the interaction between CKD and CAD. Starting with the redefinition of ischemic heart disease in 2019, the development of new methods for quantifying myocardial ischemia, and the optimization of contrast agents to minimize the occurrence of contrast-induced nephropathy and continuing with the identification of new pathophysiological mechanisms and novel biomarkers, this paper is intended to clearly illustrate the interaction between CKD and CAD and elucidate the role of cardiorenal biomarkers in this process.

## 2. The Relationship between Chronic Kidney Disease and Ischemic Heart Disease

Richard Bright, a British physician, was the first to report the existence of a relationship between chronic kidney disease and cardiovascular disease, thus establishing the concept of a renal origin of cardiovascular disease. His writings from 1836 contain the following passages relevant to this association between the heart and the kidneys: “The obvious structural changes in the heart have consisted chiefly of hypertrophy… and what is most striking, out of fifty-two cases… no valvular disease could be detected in thirty-four… This naturally leads us to look for some less local cause”, and “It is observable, that the hypertrophy of the heart seems, in some degree, to have kept pace with the advance of diseases in the kidneys; for in by far the majority of cases, when the heart was increased, the hardness and contraction of the kidney bespoke the probability of long continuation of the disease” [13].

The largest population-based study conducted by Go et al. demonstrated that the decreased glomerular filtration rate was the principal independent risk factor for cardiovascular events (CAD, chronic heart failure, and stroke), even after the risk factors had been controlled [14,15]. As we know, patients with CKD have an extraordinarily high risk of cardiovascular mortality: more than half of the deaths recorded among patients with a CKD stage of 4 or 5 are due to a cardiovascular cause (in most cases, ischemia), which can be compared to 26% for the population with normal renal function [16]. Studies indicate that there is a continuous relationship between albuminuria and cardiorenal risk in both the CKD and healthy populations. Albuminuria is considered an independent risk factor and prognostic marker, increasing the risk of mortality, independent of eGFR [8,17]. A meta-analysis involving more than 1.4 million patients revealed an association between albuminuria and cardiovascular disease. Furthermore, it was observed that the incidence of cardiovascular events decreased with the introduction of antiproteinuric measures due to the blocking of the renin–angiotensin–aldosterone system [8].

For these patients, cardiovascular mortality results from complications associated with the atherosclerotic process—stable coronary artery disease and acute coronary syndromes (ACSs), heart failures, or malignant arrhythmias, especially in the advanced stages of CKD. In more than 70 studies that included non-dialysis CKD patients, the correction of cardiovascular risk factors such as hypertension, diabetes mellitus, and dyslipidemia did not neutralize the impact of CKD on cardiovascular risk, which is directly proportional to the progression of CKD [18]. The risks of atrial fibrillation and chronic coronary syndromes (CCS) are twice as high in patients with GFR < 60 mL/min; once atrial fibrillation occurs, the risk of CKD progressing to ESKD increases approximately three times. The risk of developing heart failure is three times higher in patients with GFR < 60 mL, and, once it has appeared, it is associated with a rapid progression of CKD, recurrent hospitalizations, and ultimately death [19].

### 2.1. Common Risk Factors

Similar to CAD, the onset and progression of CKD involve various implicated risk factors. The non-modifiable factors include genetics, age, gender, and low birth weight. The modifiable factors encompass high blood pressure, which can act both as a cause and an effect, as well as proteinuria, type 2 diabetes, dyslipidemia, obesity, chronic use of analgesic medications or non-steroidal anti-inflammatory drugs, and smoking [5,6].

The specific risk factors for atherosclerosis are common to both pathologies and include the following: hypertension, insulin resistance, diabetes, dyslipidemia, and smoking. These factors contribute to cardiovascular atherosclerosis by influencing the progression of CKD, affecting large vessels (such as coronary arteries and renal artery stenosis) and small vessels (via nephrosclerosis and endothelial dysfunction) [8,20,21].

#### 2.1.1. Hyperglycemia

Hyperglycemia is strongly associated with the development and progression of both CKD and CAD. In diabetic patients, the association of the triad of hyperglycemia, insulin resistance, and hyperinsulinemia increases a patient’s cardiovascular risk two- to threefold, making mortality in this case comparable to that of individuals who have experienced a myocardial infarction [22]. According to studies, individuals with type 2 diabetes have a fivefold higher risk of developing CAD. Insulin resistance (IR), whose key mechanism is oxidative stress, is associated with most chronic complications in these patients, namely, CAD, CKD, retinopathy, liver dysfunction, and neuropathy [23,24,25]. The increase in glucose concentrations, levels of free fatty acids (FFA), and leptin levels is associated with increased production of oxygen free radicals and leads to the occurrence of the previously mentioned microvascular and macrovascular complications [25].

While diabetes induces inflammation, mitochondrial dysfunction, myocardial fibrosis, and increased oxidative stress, new hypoglycemic therapies represented by GLP-1 receptor agonists and SGLT2 inhibitors counteract the development of atherosclerosis and cardiovascular complications by reducing oxidative stress, increasing nitric oxide release, and lowering IR, hyperlipidemia, and cardiovascular risk [25]. In particular, SGLT2 inhibitors, the most recent class of drugs, are associated with a variety of pleiotropic effects that ensure nephroprotection and cardioprotection, regardless of co-affliction with diabetes mellitus. Among these effects is the antihypertensive impact, reducing systolic blood pressure by 3–6 mmHg and diastolic blood pressure by 0–2 mmHg. [26]. Empagliflozin, the most-studied representative, exerts a strong cardioprotective and nephroprotective effect by significantly reducing oxidative stress and consequently IR and the proinflammatory status [25]. Also, recent studies have shown that SGLT2 inhibitors are associated with a decrease in the progression of CKD, regardless of co-affliction with diabetes mellitus. Empagliflozin intervenes in the decline in eGFR, even in patients with eGFR levels as low as 20 mL/min per 1.73 m^2^ [27,28,29]. According to the EMPAREG study conducted on patients with concomitant CAD, the occurrence of major cardiovascular events decreased by 14%, the rate of hospitalization for heart failure decreased by 35%, and the risk of all-cause mortality decreased by 32%. When used in conjunction with standard therapy, empagliflozin can reduce the risk of GFR decline by 40% in patients with type 2 diabetes, CKD, and CAD [25,30].

#### 2.1.2. Hypertension

Affliction with hypertension among patients with CKD is one of the most important factors contributing to cardiovascular morbidity and mortality. It is important to note that the SCORE classification for cardiovascular risk assessment cannot be applied to hypertensive patients with CKD. These patients are already classified as having a very high risk due to renal impairment. The progression rate of CKD is directly proportional to the severity of hypertension. Hypertension is the second-most-important affliction leading to CKD after diabetes mellitus, and it is an independent risk factor for other cardiovascular events, namely, CAD and myocardial infarction, stroke, heart failure, and peripheral artery disease [7,31]. A meta-analysis of 18 randomized clinical trials involving 15,924 patients diagnosed with CKD demonstrated that intensive blood pressure control was associated with a reduction in all-cause mortality [7,32].

High blood pressure can be both a cause and a consequence of CKD. Intraglomerular hypertension is a consequence of the transmission of systemic arterial pressure; alternatively, it can be caused by various glomerular pathologies. Direct damage to the endothelium increases parietal stress in the glomerular capillaries, while increased mesangial pressure contributes to the initiation and progression of glomerular injury. The progression of hypertension occurs through increased sympathetic tone, heightened activity of the renin–angiotensin–aldosterone system, endothelial dysfunction (especially in the advanced stages of CKD), and increased arterial stiffness [33]. According to studies, there is a correlation between ambulatory blood pressure monitoring (ABPM) and left-ventricular mass index as well as proteinuria. ABPM monitoring has proven more effective for quantifying hypertension compared to occasional measurement. The hallmark of hypertensive patients with CKD is left-ventricular hypertrophy (LVH), which can be quantified by the observed ratio of estimated left-ventricular mass (LVM); recently, it was found that the observed ratio of predicted LVM is independently associated with increased cardiovascular events for patients corresponding to CKD stages 3–5 [14,34].

Most studies suggest that the optimal blood pressure level for patients with CKD should be <130/80 mmHg. The recent guidelines published by Kidney Disease Improving Global Outcomes (KDIGO) regarding the management of hypertensive patients state that systolic blood pressure should be <120 mmHg based on the SPRINT study [7,35], a randomized clinical trial that correlated a reduction in systolic blood pressure to <120 mmHg with a decrease in major cardiovascular events and mortality compared to a systolic blood pressure level of < 140 mmHg [7,36]. According to recent studies, two new medications are associated with known antihypertensive medications: SGLT2 inhibitors, which can reduce blood pressure by 7–10 mmHg, and GLP-1RAs, which have a more modest effect on blood pressure but can reduce it by 1–5 mmHg. The mechanism by which SGLT2 inhibitors lower blood pressure has not been fully elucidated, but it is believed to occur through osmotic diuresis, leading to the elimination of water and glucose in the urine, resulting in decreased plasma volume, weight loss, and effects on blood vessels (vasodilation, increased nitric oxide synthesis, and reduced inflammation) [7,37].

#### 2.1.3. Dyslipidemia

Patients with CKD exhibit a characteristic lipid profile characterized by a combination of hypertriglyceridemia with low levels of HDL cholesterol and, in most cases, normal levels of low-density lipoprotein cholesterol. The consequences include dyslipidemia and the accumulation of atherogenic particles [14]. The key role of HDL-cholesterol lies in participating in the reverse transport of cholesterol from peripheral tissues to the liver. Additionally, it prevents the oxidation of LDL-cholesterol by reactive oxygen species (ROS) and shields the endothelium from the harmful effects of oxidized LDL particles. Studies have highlighted the ability of HDL-cholesterol to induce the production of nitric oxide by endothelial cells and to exert antiapoptotic and anti-inflammatory effects [38].

According to recent studies, CKD causes a decrease in HDL levels due to impaired maturation of HDL and post-translational modifications. This decrease is associated with an atherogenic profile characterized by the loss of antiatherosclerotic properties, a decline in the metabolism of triglyceride-rich lipoproteins, a slower rate of apoA-I and A-II synthesis via the liver, and enhanced activity of cholesteryl ester transfer protein (CETP) [38,39,40]. These changes share a commonality in the presence of a uremic environment: the progression of oxidative stress and chronic inflammation. Most studies conducted over time have highlighted that in CKD, the corresponding increased cardiovascular risk is not solely associated with lipoprotein levels but is also determined by the quality of the lipoproteins. For instance, in the 4D study (Die Deutsche Diabetes Dialyze Studie), a reduction in LDL l in CKD patients undergoing hemodialysis through treatment with atorvastatin did not influence cardiovascular mortality. Similarly, in the PREVEND IT study (Prevention of Renal and Vascular End-stage Disease Intervention Trial), a reduction in cardiovascular mortality was not observed among CKD patients with microalbuminuria treated with pravastatin [38,41,42].

### 2.2. Inflammation-Oxidative Stress-Endothelial Dysfunction Triad

Inflammation is a key process observed in patients with CKD, characterizing this pathology as a multicausal systemic inflammatory disease. As kidney function declines, a variety of inflammatory mediators accumulate. The pro-inflammatory state is usually determined by several factors, including infections (such as periodontal disease), oxidative stress, metabolic acidosis, low clearance of pro-inflammatory cytokines, insulin resistance, post-translational modifications of blood lipoprotein molecules, and genetic background [8,43,44]. The chronic systemic pro-inflammatory status induced by CKD contributes to vascular and myocardial remodeling processes, the generation and progression of atherosclerotic lesions, vascular calcification, myocardial fibrosis, and extensive valvular calcifications. All these pathological changes lead to accelerated and premature aging of the cardiovascular system [8]. In accordance, the CANTOS study (Canakinumab Anti-Inflammatory Thrombosis Outcome Study), which included approximately 10,000 stable post-myocardial infarction patients with high-sensitivity C-reactive protein, demonstrated the benefit of inhibiting the pro-inflammatory effector molecule interleukin-1β (IL-1β) using the antibody canakinumab. This benefit was more pronounced in patients with eGFR levels < 60 mL/min/1.73 m^2^ than in those with eGFR levels > 60 mL/min/1.73 m^2^ [8,45].

In CKD, endothelial function is influenced by angiotensin II (through stimulating the production of superoxide, IL 6, and endothelin), the low bioavailability of nitric oxide, and the low activity of renalase (an enzyme produced by the kidneys that governs the inactivation of catecholamines) [10]. In patients with CKD and cardiovascular impairment, it has been demonstrated that there are increased concentrations of vasopressin and endothelin-1. Endothelin-1 is an inflammation mediator with profibrotic and vasoconstrictive properties that stimulates the synthesis of transforming growth factor β (TGF-β) and nuclear factor κB (NF-κB), thereby maintaining the inflammatory status in the kidneys [29]. However, angiotensin II receptors are also found in the heart, where the release of TGF B1 and endothelin-1 from cardiac fibroblasts leads to left-ventricular hypertrophy (LVH) [29,46]. Elevated levels of CRP and IL-6 in plasma have been observed in patients with CKD and left-ventricular systolic dysfunction [29,46,47]. Additionally, the triad represented by increased CRP, IL-6, and TNF has been associated with a very high risk of mortality due to acute myocardial infarction [48].

A study conducted by Freise et al. showed that tumor necrosis factor (TNF)- and interleukin 10 (IL-10)-mediated inflammatory processes impacted pathobiological responses in the arteries of children with CKD, correlating with myocardial remodeling and coronary impairment [29,46,49]. Concurrently, hypoxia and hypoperfusion secondary to cardiovascular impairment accentuated oxidative stress in the kidneys, resulting in decreased nitric oxide bioavailability and heightened endothelial dysfunction [29]. Oxidative stress exacerbates the inflammatory response through the activation of proinflammatory cytokines (IL-1, IL-6, and TNF-α), and the resultant chronic inflammation leads to the progressive loss of renal function [50].

Epicardial adipose tissue (EAT) constitutes the true visceral fat depot of the heart, accounting for approximately 20% of the heart’s weight and covering 80% of the cardiac surface, often along the coronary arteries [14,51,52,53]. Recent studies have demonstrated that EAT can act as an active organ and produce bioactive adipokines and proinflammatory and proatherogenic cytokines, such as TNF-α, monocyte chemotactic protein (MCP-1), IL-6, and resistin. This contributes to premature cardiovascular disease and consequently CAD, thus increasing mortality, especially among ESKD patients [14].

### 2.3. Atherosclerosis and Vascular Calcifications

Atherosclerosis is a pathological process characterized by the thickening and stiffening of the arterial wall, secondary to the formation of atheroma plaques in the inner layer of the vessels, thereby impeding blood flow to organs and tissues. Coronary atherosclerosis, also known as CAD, poses the greatest risk of cardiovascular damage in patients with kidney problems. In contrast to the general population, individuals with a known chronic kidney disease (CKD) are subject to the involvement of a series of new mechanisms specific to renal pathology, in addition to traditional risk factors. These include endothelial dysfunction, oxidative stress, chronic inflammation, bone metabolism disorders (hyperphosphatemia, hyperparathyroidism, and vascular calcifications), and the impact of uremic toxins. The accumulation of homocysteine, for instance, exerts an atherogenic effect through the direct oxidation of lipoproteins [8,54,55]. Autopsy studies of patients with CKD have revealed more-advanced atherosclerotic plaques and a higher prevalence of calcified plaques [56].

Arterial calcification is very common for patients with CKD and becomes more significant as renal dysfunction worsens. Vascular calcification can develop in the intimal or medial layer of a blood vessel. While, in the general population, it most often occurs in the intimal layer, in the case of CKD patients, calcification of the medial layer is characteristic; also known as “Monckeberg sclerosis”, it is associated with intimal calcification. [14,57]. The hemodynamic consequences of this process include a decrease in coronary microcirculation and arterial elasticity, an increase in pulse wave velocity, and an increase in left-ventricular hypertrophy. All these changes contribute to CAD and heart failure [14,58,59]. Medial calcification is characterized by diffuse calcification of muscular arteries in a circumferential pattern and alteration of their contractile phenotype, while intimal calcification is associated with inflammation and focal occlusion secondary to the formation of atheroma plaque [14]. A meta-analysis of 47 studies, including patients with various stages of CKD and transplanted patients, identified a common prevalence of coronary artery calcifications across CKD stages amounting to ~60%, with an associated nearly two- to fourfold increase in mortality and cardiovascular events [60,61].

The progression of the calcification process in CKD is attributed to increased exposure to calcium and phosphorus due to bone metabolic disorders and the imbalance between factors that promote calcification (receptor activator of nuclear factor-κB and receptor activator of nuclear factor-κB ligand) and factors that inhibit calcification (klotho, osteoprotegerin, and fetuin A) [60,62,63]. Furthermore, CKD disrupts the metabolism of vitamin K, leading to an additional reduction in calcification inhibitors. When vitamin K antagonists (acenocoumarol and warfarin) are utilized to treat CKD patients, they exacerbate the calcification process, contributing to extensive vascular and valvular calcifications in these patients [60,64,65,66].

Malnutrition–inflammation–atherosclerosis/calcification (MIAC) syndrome constitutes the association between increased levels of pro-inflammatory cytokines, malnutrition, and atherosclerosis/calcification in ESKD patients [14]. Stenvinkel et al. hypothesized that inflammation, atherosclerosis, and malnutrition cause a vicious circle, in which pro-inflammatory cytokines play a central role. This syndrome is associated with increased mortality and morbidity among affected patients [14,67].

Coronary artery calcification can be detected from the first years of dialysis, reflecting the severity of atherosclerotic damage and an increased risk of coronary events. A recent study on hemodialysis patients, which correlated coronary artery calcification score (CACS) with coronary flow velocity reserve (CFR), demonstrated that the functional deterioration of coronary arteries was initiated by a low CACS. As expected, patients with a CACS above 10 had a significantly lower CFR [14,68]. Evidence from invasive coronary angiography has revealed that patients with moderate to severe CKD have a fourfold increased risk of multivessel CAD compared with those with early-stage CKD, even after diabetes control [56,69]. Autopsy studies performed on patients with advanced CKD and undergoing hemodialysis revealed numerous extensive and calcified atherosclerotic lesions. The progression of this process is the result of accelerated atherosclerosis and lipoprotein changes, associated with the preponderance of risk factors for calcification and the accumulation of toxic metabolites (N—trimethylamine oxide) [56,60]. Thus, there is likely to be a reciprocal relationship between the coronary atherosclerosis process and renal function in which CAD is associated with worsening renal dysfunction and vice versa. Sanchis et al. proposed the term “inflammaging” to conceptualize the process of premature vascular senescence resulting from metabolic alterations and a chronic proinflammatory status [56,70].

### 2.4. Uremic Cardiomyopathy

Myocardial fibrosis and left-ventricular hypertrophy (LVH) are common components of uremic cardiomyopathy. LVH is found in one out of three patients with CKD, reaching 80% for patients with ESKD; LVH is an independent predictor of mortality for patients with CKD, regardless of stage [8,71]. Three factors are implicated in the pathogenesis of LVH. Afterload is negatively influenced by abnormal arterial stiffness, increased systemic arterial resistance, and systolic arterial hypertension; continuous left-ventricular overload induces maladaptive changes, leading to myocyte death, resulting in left-ventricular eccentric hypertrophy, systolic dysfunction, and a decreased ejection fraction [8,72]. The role of preload in LVH pathology is manifested through intravascular volume expansion, causing volume overload, myocyte length expansion, and subsequent eccentric or asymmetric remodeling of the left ventricle [8,73].

LVH contributes to reducing coronary reserve, which is already compromised by the decreased synthesis of endothelial nitric oxide from the early stages of the disease. This fibrosis type is characterized by the diffuse deposition of collagen between capillaries and cardiomyocytes, funneling into maladaptive ventricular hypertrophy with subsequent heart dilatation [8]. LVH leads to a reduction in the coronary reserve, which is already affected by the decrease in endothelial nitric oxide synthesis from the early stages of the disease. The high prevalence of LVH and the potential occurrence of malignant arrhythmias could partly explain the elevated rate of sudden cardiac death among patients [10].

Studies indicate a correlation between CKD and aortic or mitral valve disease, with heart valve damage associated with a poor prognosis among these patients. In early stages (G1–G3), isolated calcifications of valves and coronary arteries are observed. Significant valvular calcification occurs in stage G5 in at least 88% of patients. Starting from stage G3, valvular destruction occurs at a rate 10 times higher compared to patients without CKD [8,74]. The progression of valvular disease in CKD is accelerated by comorbidities such as diabetes, hypertension, anemia, infections (including infective endocarditis), malnutrition, and disorders of phosophocalcium metabolism [8,75]. Electrolyte imbalances such as hypomagnesemia are common in CKD patients and represent a potential target for CAD management. Magnesium has been observed to exert an inhibitory effect on vascular calcification by interacting with hydroxyapatite crystals, succeeding in halting the progression of vascular calcifications in advanced CKD [8,76,77].

### 2.5. Acute Coronary Syndrome in CKD Patients

Patients with CKD are more likely to have an acute coronary syndrome (ACS) rather than a chronic coronary syndrome (CCS) as the primary reason for their initial emergency hospitalization. CKD and ESKD can disrupt the classic symptoms of CAD, leading to atypical clinical presentations. Most patients are paucisymptomatic; only 44% of those with CKD G3A experiencing acute myocardial infarction (AMI) report classic symptoms, which can be compared to 72% for patients with preserved renal function. Dyspnea at rest is often the sole symptom [56,76]. Intradialytic hypotension and myocardial stunning are hemodialysis-specific syndromes that are associated with mortality and unique to dialysis patients [56]. Many acute coronary syndromes in CKD patients are of the non-ST-segment elevation (N-STEMI) form. The high incidence of N-STEMI and the prevalence of heart failure with a preserved ejection fraction suggest a distinct process from classical atherosclerotic coronary plaque rupture, with subsequent myocardial damage and diastolic dysfunction. In this case, cardiac microcirculatory dysfunction is implicated, leading to a decrease in coronary flow reserve and general capillary density [56,77,78].

Considering LVH, ECG changes are often non-specific, especially in patients with advanced CKD and ESKD. Cardiac troponin levels are frequently elevated in advanced CKD syndrome in the absence of acute coronary syndrome. A normal troponin value may rule out myocardial infarction, while elevated values are less specific [56]. According to the Acute Catheterization Strategy and Urgent Intervention Triage (ACUITY) study, CKD patients with high baseline troponin levels had significantly higher rates of death, myocardial infarction, or unplanned revascularization at 30 days and 1 year compared with those of CKD patients without initial elevation of troponin [14].

### 2.6. Uremic Toxins

In the case of CKD patients, impaired renal excretion leads to the accumulation of uremic toxins. While their role is still under research, it is well-established that these toxins are considered promoters of cardiac remodeling, vascular lesions, and renal damage [29]. The accumulation of uremic toxins begins in the early stages of CKD [79]. According to studies, there are substances that directly induce cardiotoxicity, such as indoxyl sulfate and p-cresyl sulfate. These exert endothelial cytotoxic effects in ESKD patients by generating reactive oxygen species, leading to inflammation and ultimately fibrosis [29,80].

The most well-known toxins include asymmetric dimethylarginine (ADMA), advanced glycation end products (AGEs), and trimethylamine N-oxide (TMAO) [29,81]. ADMA contributes to endothelial dysfunction by strongly inhibiting the synthesis of nitric oxide, reducing cardiac output, and increasing systemic vascular resistance, consequently elevating blood pressure. [10]. Elevated concentrations of ADMA and sympathetic hyperactivity are strongly associated with left-ventricular hypertrophy [10]. Furthermore, ADMA is implicated in the onset of renal anemia. Circulating AGEs, due to their inability to break down, accumulate in the heart and kidneys, causing tissue damage and inflammation. TMAO is a promoter of fibrosis, and elevated levels are correlated with accelerated atherosclerosis and a poorer prognosis among patients with chronic heart failure [29,80].

CKD is now recognized as an independent risk factor for CAD. According to studies, decreased GFR and proteinuria have been independently associated with CAD. The risk of CAD increases in parallel with a decrease in GFR, placing patients with ESKD at the highest risk [14]. The basic physiopathological mechanisms of CAD in CKD patients are represented by the association of traditional risk factors with those specific to CKD patients, i.e., chronic inflammation, oxidative stress, and hyperparathyroidism [14]. Future diagnostic strategies may involve early screening for subclinical atherosclerosis in CKD patients, using a variety of parameters such as carotid intima–media thickness, pulse wave velocity, aortic and brachial augmentation indexes, the ankle-brachial index, or aortic systolic blood pressure [82].

## 3. Cardiorenal Biomarkers

Biomarkers are signals produced by the body that reflect the functionality of specific organs and systems. They are used in clinical practice to establish diagnoses and prognoses and assess the severity of pathologies or guide therapy. Biomarkers come in the form of hormones, proteins, or enzymes with concentrations that vary according to certain physiological or pathological conditions. For a biomarker to be useful in clinical practice, it must meet specific criteria: it should appear in the early stages of the disease, be quantifiable, predict disease severity, aid in risk stratification, be a reliable prognostic indicator, exhibit high sensitivity and specificity, guide treatment decisions, and be cost-effective [83].

Various clinically available cardiac biomarkers, alongside recently discovered ones, are associated with the risk of both CKD and cardiovascular damage. In a large cohort of diagnosed CKD patients, cardiac markers like N-terminal prohormone of brain natriuretic peptide (NT-proBNP), high-sensitivity troponin I (hsTn I), plasma growth differentiation factor 15 (GDF-15), and soluble suppression of tumorigenicity 2 (sST2) were significantly linked to an increased risk of developing atherosclerotic disease and heart failure [83,84]. Syndecan-1 has emerged as another promising dual cardio-renal biomarker, as there is solid evidence about its role in glococalyx injury and the subsequent endothelial dysfunction. One recent study even highlighted the potential role of syndecan-1 in predicting the development of acute kidney injury in patients with acute heart failure [85,86,87]. There is an overlap of risk factors for CKD and cardiovascular disease (such as hypertension, diabetes, and smoking). However, CKD has proven to be an independent risk factor for fatal and nonfatal cardiovascular disease events [15,88,89]. For instance, the inflammation and fibrosis biomarker Galectin-3 plays a causal role in both cardiac and renal fibrosis, representing a potential common element in both CKD progression and cardiovascular damage [88]. CKD, especially in advanced stages, is characterized by an “uraemic milieu” associated with unique risk factors. Hyperphosphatemia leads to a depletion of Klotho levels and increased FGF-23 levels, both of which directly contribute to left-ventricular hypertrophy followed by cardiac fibrosis and coronary calcification [88,90].

**Figure 1:**
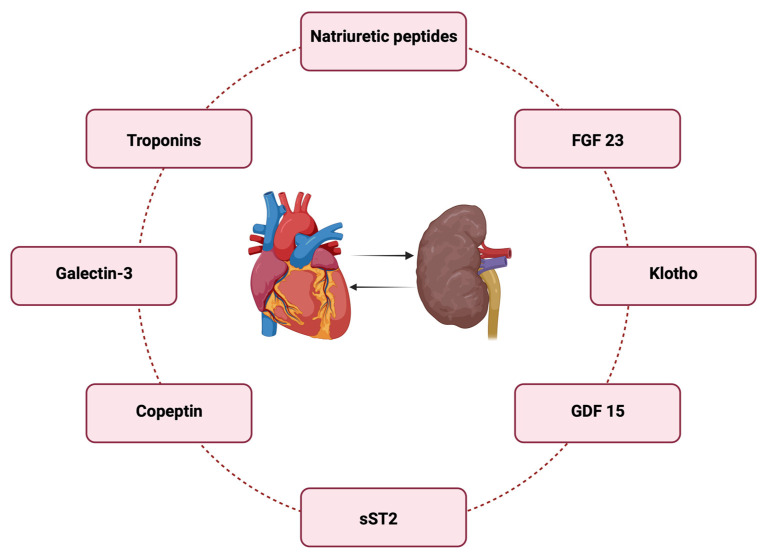
Cardiorenal biomarkers. This image was created with BioRender.com (accessed on 18 November 2023).

In this context, we will analyze a series of common biomarkers for both chronic renal and ischemic heart pathology. We will start with classic biomarkers frequently used in clinical practice and then explore some new biomarkers with increased potential for use, although they may not yet be included in guidelines and daily practice (see Figure 1).

### 3.1. Cardiac Troponins

Troponins are a family of proteins present in all striated muscles, serving as the primary mediators of muscle contraction by linking the thick filaments (myosin) to the thin ones (actin and tropomyosin) of the sarcomere through calcium binding [91]. Cardiac troponin consists of three subunits, cTnC, cTnI, and cTnT, of which only the latter two are utilized in clinical practice. While small amounts of cTnT are found in skeletal muscle, cTnI is exclusively found in the heart, making it the most specific troponin subunit for cardiac muscle [92].

Cardiac troponin serves as the most reliable biomarker for ACS, with its increased serum level forming a crucial component of the acute myocardial infarction diagnostic protocol as per European and American guidelines. Detectable in the circulation 3–12 h after myocardial injury, its concentration correlates directly with the extent of the injury [93]. cTnT and cTnI serve as potential biomarkers for clinically diagnosing myocardial damage in various conditions (chronic heart failure, acute hypotension, left-ventricular strain, pulmonary embolism, hypertrophic cardiomyopathy, myocarditis, or exposure to toxic substances) and non-cardiac pathologies (sepsis, stroke, CKD, hemodialysis, and diabetes mellitus) [83,92]. Moreover, at least five mechanisms contribute to the release of cardiac troponin from cardiomyocytes, excluding myocyte necrosis from ACS: membrane blebs, increased membrane permeability, proteolysis, apoptosis, and normal cell turnover [83,94].

Normally, troponin is located inside myocytes in the cytoplasm and is released into the circulation when the cell membrane is damaged. In patients with CKD, circulating cardiac troponin levels are often elevated, even in the absence of ACS. Elevated cardiac troponin levels result from continuous myocardial injuries caused by prolonged exposure to uremic toxins, left-ventricular hypertrophy, silent plaque rupture in the presence of diffuse coronary atherosclerosis, apoptosis of cardiomyocytes, and chronic heart failure [7,93,95]. This may explain why patients with troponin concentrations above the upper limit have a twofold higher risk of subsequent myocardial infarction or cardiac-related death within one year, regardless of the diagnosis [7,96,97]. A recent study that included patients presenting to the hospital for suspected ACS found that elevated levels of high-sensitivity cardiac troponin (hs-cTn) increased with a decline in renal function. Thus, from 10% in patients with normal renal function, it reached 66% in patients with eGFR levels < 30 [7,98]. Current estimates indicate that only 2–20% of the CKD population undiagnosed with cardiovascular disease have undetectable troponin levels at baseline [99].

A second hypothesis, recently proposed to elucidate this chronic elevation in troponin levels, involves a reduction in renal clearance. While the molecular weight of intact troponins hinders passive renal clearance, smaller degradation products of troponin might undergo renal filtration. [95]. Thus, it is conceivable that these smaller cTn fragments accumulate in CKD patients and are detectable through specific tests. A study involving 63 hemodialysis patients revealed that immunoreactive troponin fragments ranging from 8 to 25 kDa in size could be identified in the blood, suggesting their potential passage through the renal filter due to their small size [100,101]. Another study, encompassing 24 patients with varying renal function, demonstrated that urinary cTnT was not detectable in patients with normal renal function after myocardial injury, but it was detected in ESKD patients, hinting at the existence of a tubular component in excretion [100,102].

Several studies have scrutinized the prognostic implications of detectable cardiac troponin levels in CKD patients for whom affliction with ACS has been excluded. Most have shown that heightened concentrations of cardiac troponins are linked to increased all-cause mortality, particularly that stemming from cardiovascular causes. Sandoval et al. observed that a change above the estimated biological variability level measured with hs-cTnI and hs-cTnT assays at a 3-month interval was associated with a higher risk of death. For hs-cTnI, there was also an association with sudden cardiac death [88,103]. The Chronic Renal Insufficiency Cohort (CRIC) study comprised 3243 subjects, with 84% exhibiting elevated levels of cardiac troponin strongly correlated with left-ventricular hypertrophy. Within this group, an increase in CRP, LDL/total cholesterol, phosphate, and FGF-23 levels was noted, along with a decrease in HDL, albumin, and hemoglobin levels. At a 6-year follow-up, 95% of these patients also developed heart failure [100]. Another study aimed to explore the presence of occult obstructive CAD in 142 patients with elevated cTnT levels upon the initiation of renal replacement therapy. Of the 60 asymptomatic patients evaluated, 35 had obstructive CAD, and 27 had multivessel CAD, underscoring that cTnT is an independent predictor of asymptomatic multivessel CAD [93].

The diagnosis of ACS for a patient with CKD and elevated troponin values can be challenging in the absence of symptoms or ECG changes suggestive of acute ischemia. Nevertheless, serial determinations prove as effective as they are when used for patients without renal impairment, according to studies. According to the fourth universal definition of myocardial infarction, cardiac troponin values above the upper reference limit of the 99th percentile denote myocardial injury, with the injury being deemed acute in the presence of an increase, decrease, or both [91,104,105]. A meta-analysis of 14 studies indicated that the specificity of elevated troponin levels above the 99th percentile significantly diminishes in patients with CKD. Therefore, an accepted recommendation is that troponin-positive enzyme swings occur at least 20% of the time in testing series within less than 9 h, a hypothesis developed by The National Academy of Clinical Biochemistry [91,106].

Consequently, cardiac troponins, as biomarkers for ischemic cardiac damage, have demonstrated utility both in ACS, subject to concentration changes during serial testing, and in chronic coronary damage. Elevated cardiac troponins, found in over half of CKD patients regardless of the stage, are associated with a poor prognosis and an increased risk of all-cause mortality, particularly cardiovascular events. In pre-dialysis patients, elevated cardiac troponin levels serve as an independent predictor of asymptomatic multivessel coronary disease.

### 3.2. Natriuretic Peptides

Natriuretic peptides constitute a family of hormones, including ANP (atrial natriuretic peptide), BNP (brain B-type natriuretic peptide), and CNP (C-type natriuretic peptide). They play a crucial role in sodium homeostasis and body volume regulation, overseeing processes such as natriuresis, vasodilation, and diuresis to safeguard the cardiovascular system from the effects of volume overload [93,107]. BNP and NT-proBNP stand out as the most relevant biomarkers, and they have also been introduced in international guidelines. BNP is synthesized by the ventricular myocardium in response to parietal stress induced by volume/pressure overload and myocardial ischemia, initially in the form of precursors (pre-proBNP and proBNP). BNPs, the active metabolites, exert vasodilatory and diuretic effects, which reduce left-ventricular load, while NT-proBNP, the inactive metabolite with a longer half-life, results from the cleavage of the proBNP precursor [92,93,108]. They play a crucial role in the diagnosis and prognosis of both acute and chronic heart failure.

Moreover, elevated levels of natriuretic peptides in the blood can occur as a secondary response to various conditions such as myocardial ischemia, pulmonary embolism, tachyarrhythmias, stroke, CKD, and liver or thyroid damage. The increase in natriuretic peptides during myocardial ischemia is considered a compensatory mechanism, playing a protective role against neutrophil-induced endothelial damage. This involves suppressing the adhesion of neutrophils to the endothelium, downregulating the expression of CD18 on neutrophils, and mitigating the release of elastase. These changes collectively aim to limit the area of myocardial necrosis [109]. A series of studies revealing a relationship between NT-proBNP and CAD have been conducted. In a study involving 94 patients with stable angina undergoing coronary angiography, Weber et al. demonstrated that an elevated NT-proBNP level is an independent predictor of obstructive CAD [110,111].

The serum levels of natriuretic peptides, particularly NT-proBNP, are influenced by renal function. Studies have demonstrated a direct proportional relationship between an increase in NT-proBNP levels and a decrease in GFR, especially in patients with chronic heart failure [29]. The mechanisms of this connection are not fully understood, but it is considered that in patients with CKD, natriuretic peptide levels can be elevated due to impaired renal excretion [112,113]. In this scenario, a high level of BNP/NT-proBNP is associated with an increased risk of accelerated progression from CKD to ESKD [114]. However, studies indicate that the increase in NT-proBNP is associated with a negative prognosis. In patients with CKD, especially ESKD, natriuretic peptides may be elevated to approximately 10–100 times their normal range [88]. Additionally, recent studies have highlighted the role of B-type natriuretic peptides as markers for the early detection of renal involvement in ventricular stress [115]

Multiple studies have confirmed the utility of both BNP and NT-proBNP as markers in assessing cardiovascular risk among patients with CKD. In a study involving 213 patients with CKD (up to stage G4), Vickery et al. demonstrated that an increased value of NT-proBNP, associated with a higher level of hsCRP, was independently linked to increased mortality from all causes [93,116]. In another study of hemodialysis patients, NT-proBNP levels were elevated in all patients, and cardiac troponin was elevated in approximately 40% of them. Both NT-proBNP and troponin elevation were strongly associated with cardiovascular disease, especially CAD, with approximately 30% of patients dying from acute myocardial infarction [93,117].

In conclusion, we can assert that natriuretic peptides, especially BNP and NT-proBNP, play a central role in cardiovascular and renal pathology through the involved pathophysiological mechanisms. Although they are well-established for their role as ‘gold standard’ biomarkers in the prognosis, evolution, and treatment of acute and chronic heart failure, according to international guidelines, their involvement in the prognosis and evolution of patients with coronary disease is becoming increasingly evident.

### 3.3. Galectin-3

Galectins constitute a family of β-galactoside-binding lectins expressed widely across human tissues, including immune, epithelial, and endothelial cells and sensory neurons. They play crucial roles in diverse biological processes such as cell proliferation and differentiation, inflammation, fibrogenesis, angiogenesis, and host defense [118,119]. Galectin-3, in particular, has been correlated with numerous cardiovascular risk factors and exhibits the ability to bind to von Willebrand factor, implicating it in the modulation of early thrombus formation [120,121].

Elevated serum levels of galectin-3 have been identified in nearly all cardiovascular conditions, showcasing both prognostic and diagnostic value in some instances. In heart failure, gelectin-3 serves as a marker of fibrosis and inflammation, playing a pivotal role in the condition’s development and progression, thereby predicting increased morbidity and mortality. A 26-month study involving patients with chronic heart failure and CAD revealed that galectin-3 values > 21 ng/mL were associated with elevated mortality risk, establishing galectin-3’s independence as a predictor for mortality from all causes and rehospitalization [118,122]. In patients with a high cardiovascular risk undergoing coronary angiography, galectin-3 emerged as an independent predictor of the risk of cardiovascular death, with higher galectin-3 expression associated with multivessel [123]. Notably, increased serum galectin-3 levels were indicative of heightened myocardial fibrosis [118,124]. Notably, extensive long-term studies identified galectin-3 as a strong and independent predictor of cardiovascular mortality in CAD patients over the course of approximately seven years [123]. In acute myocardial infarction, galectin-3 increase showed a direct proportional relationship with other biomarkers (matrix metalloproteinase-3, monocyte chemoattractant protein-1, and interleukin-8) and was positively associated with infarct size and ventricular remodeling in patients with a history of complicated myocardial infarction [125]. Elevated levels of this biomarker in patients with ACS were found to be correlated with a reduction in left-ventricular ejection fraction and GFR [126].

Due to its reflection of inflammation and fibrosis, galectin-3 serves as a valuable biomarker for assessing both cardiac and renal function, establishing its role as a cardiorenal biomarker. Studies involving heart failure patients have shown that increased galectin-3 levels are indicative of heightened neurohumoral activity and reduced GFR. Additionally, independent associations have been found between galectin-3 levels and microalbuminuria [118,127]. Renally, galectin-3 serves as a biomarker providing insights into the pathophysiological processes underlying renal dysfunction and the progression of CKD in individuals with cardiovascular diseases [112].

Galectin-3 is implicated in renal fibrosis and dysfunction. Notably, elevated serum galectin-3 levels correlate with GFR, with higher levels associated with an increased risk of CKD development. A 2450-participant Offspring study revealed that patients with high galectin-3 levels and apparently preserved renal function experienced a greater decline in GFR during a 3-year follow-up, suggesting that this biomarker may prove useful in the early detection of renal dysfunction [112]. Recent studies have reported that the increased level of galectin-3 in hemodialysis patients substantially increases their risk of death from cardiac causes [83]. Other studies have shown that a value of galectin-3 above 23.73 ng/mL is an independent predictor of mortality in this group [118]. Additionally, elevated serum levels of this biomarker were correlated with left-ventricular hypertrophy, independent of its geometry, suggesting its potential use in the diagnosis of early diastolic cardiac dysfunction [118,128,129]. In addition, circulating levels of galectin-3 have been reported to be a potential predictor of abdominal aortic calcification in hemodialysis patients [130]. Recently, Hsu et al. reported that the association of increased galectin-3 with hsCRP correlates with indices of vascular reactivity, suggesting a role for galectin-3 as a potential biomarker of endothelial dysfunction in CKD [119].

Therefore, we can conclude that galectin-3 is a potential valuable cardiorenal biomarker, representing the common denominator in both the pathophysiology of chronic renal damage and chronic cardiovascular damage, playing a central role in the pathological processes of fibrosis and inflammation. Its role in stabilizing the prognosis of patients with cardiorenal syndrome has been demonstrated. With the development of optimal methods for detection and application in clinical practice, it could represent a therapeutic approach to delaying the fibrotic process associated with the previously mentioned conditions, especially since it is a stable biomarker that is not associated with age or body mass index.

### 3.4. Soluble Suppression of Tumorgenicity 2 (sST2)

**Figure 2:**
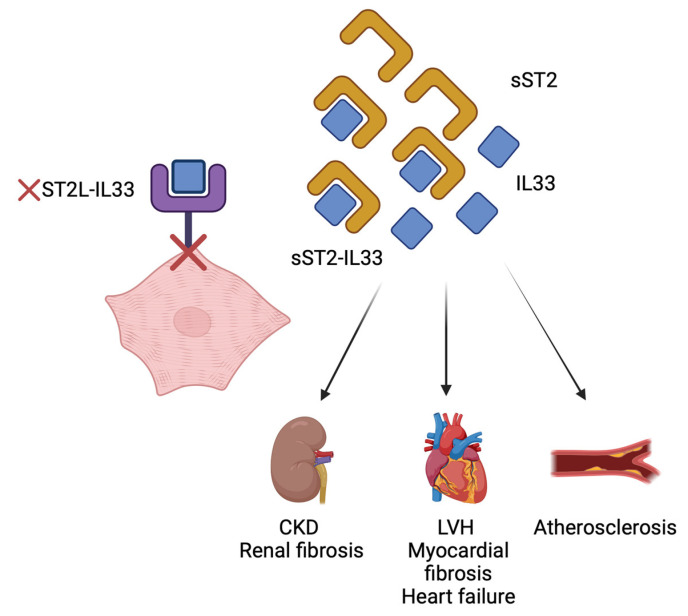
Inhibition of ST2L-IL33 results in the sST2-IL33 interactions and effect on cardiorenal axis. This image was created with BioRender.com (accessed on 18 November 2023).

Suppression of tumorigenicity 2 (ST2) is a member of the interleukin 1 (IL-1) receptor family [131]. The ST2 gene encodes two main protein isoforms: the transmembrane receptor (ST 2L) and the soluble receptor (sST 2). The interaction between ST 2L and IL-33 mediates anti-inflammatory and antifibrotic effects [131,132]. On the other hand, sST 2 competes with ST 2L for IL 33 binding; this fact increases the likelihood of the development of myocardial fibrosis and cardiac hypertrophy and remodeling and finally progression to heart failure (see Figure 2) [131,133,134]. According to studies, sST2 has been documented to serve as a predictor for mortality in heart failure. Since it appears to be less influenced by other patient characteristics such as age, body weight, anemia, and renal impairment, sST2 may offer additional information when combined with natriuretic peptides in heart failure patients. The ACC/AHA guidelines have included sST2 in a multi-marker approach for prognosis evaluation [92].

The role of sST2 in CAD has been well-documented in recent years. Its interaction with IL-33 at the level of atherosclerotic plaque influences cells involved in inflammation, thereby contributing to the progression and instability of these plaques. Pfetsh et al. observed a strong correlation between elevated levels of sST2 and certain inflammatory markers (hsCRP, IL-6) associated with chronic inflammation and fibrogenesis [131,135]. Previous studies have demonstrated a strong association between elevated levels of sST2 and increased mortality due to major cardiovascular events, including acute myocardial infarction and stroke [136]. Additionally, Zhang et al. revealed that plasma sST2 levels were significantly higher in patients with acute coronary syndrome (ACS) and complex lesions compared to those with simple lesions. This suggests that sST2 could serve as a novel marker for assessing the stability and complexity of atherosclerotic plaques [131,137]. In the context of stable coronary artery disease (CAD), several studies, including KAROLA, have proposed that elevated sST2 levels independently predict long-term mortality in this patient population. Furthermore, numerous studies that were conducted on patients with chronic ischemic heart disease who experienced an acute myocardial infarction and that were assessed through imaging methods have indicated that there is a direct relationship between increased sST2 levels and left-ventricular remodeling [131]. Moreover, several studies have identified a correlation between sST2 levels and circulating aldosterone, indicating an interconnected relationship between the two pathophysiological pathways. Currently, ongoing research is exploring the role of the IL-33/sST2 system in the pathogenesis of post-myocardial infarction cardiac remodeling [131,138].

Renal fibrosis typically manifests in the advanced stages of CKD, characterized by renal interstitial fibroblastic hyperplasia and excessive extracellular matrix deposition, ultimately leading to the destruction of renal tubular and interstitial structures [139]. In conjunction with cardiac damage, the sST2–IL-33 complex has been identified as a key driver of renal fibrosis and inflammation. Gungor et al. observed elevated IL-33 and sST2 levels with progressive CKD stages, and studies indicate that patients with increased sST2 levels have poorer prognoses [139]. Homsak et al. demonstrated that sST2, serving as a marker of fibrosis and remodeling, can be used to stratify CKD patients, particularly those with ESKD, based on their risk of all-cause mortality [132]. Combined with NT-proBNP, it enhances risk assessment, with the highest risk observed in individuals with elevated values of both biomarkers [132,138]. Therefore, it has been hypothesized that when multiple biomarkers are used, sST2 adds value to effective patient monitoring, potentially allowing better assessment of the risk of death among CKD patients, especially ESKD under substitution treatment [140]. Notably, unlike NT-proBNP or galectin-3, sST2 is independent of hemodialysis, exhibiting low biological variability, supporting its potential routine use in follow-ups of ESKD patients, irrespective of the timing of sample collection in relation to the hemodialysis session [132,141].

Therefore, sST2 stands as a crucial biomarker, intertwining with vital pathophysiological mechanisms (inflammation and fibrosis), which have been suggested to be fundamental factors in the progression of both renal and cardiac chronic damage. It emerges as an independent prognostic marker in renal and cardiovascular pathology. While numerous studies have explored its impact on cardiovascular pathology, ongoing research is delving into its involvement in nephrological disease, investigating the pathophysiological pathways it forms alongside IL-33.

### 3.5. Copeptin

Copeptin, a glycopeptide co-secreted with arginine-vasopressin (AVP) from the same precursor (prepro-hormone AVP), is released into the circulation in equimolar amounts with AVP. Specifically, it reflects the amount of AVP in plasma or serum [142,143]. Copeptin’s roles are not fully understood, but it is hypothesized to contribute to the correct structural formation of AVP. Both peptides are released in response to similar stimuli, such as increased plasma osmolality or decreased plasma volume, pain, nausea, or pregnancy. Copeptin has proven useful in establishing diagnoses of diabetes insipidus and serves as a prognostic marker in various conditions such as sepsis, shock, cardiovascular events, pulmonary disease, and renal disease. It is currently used as a surrogate marker for AVP due to its stability in serum and plasma as well as its longer half-life than AVP [142].

The cardiovascular biomarker qualities of copeptin have been extensively studied in various clinical cases, particularly in the diagnosis and prognosis of myocardial infarction and CAD, as well as in the diagnosis and prognosis of heart failure. These studies have shown promising results [144]. The first study highlighting the biomarker role of copeptin was conducted in 2009 by Reichlin et al. It revealed that copeptin was detectable in the circulation at large quantities before cardiac troponins could be detected in patients with myocardial infarction [145]. In a recent study involving 271 patients with typical symptoms of ACS, it was found that among CK-MB, hsTnI, and copeptin, the last item proved superior to hsTnI in diagnosing patients with chest pain within the first two hours of onset [146]. In heart failure, multiple studies have demonstrated that copeptin outperforms natriuretic peptides in predicting mortality and short/long-term prognosis. This superiority may be attributed to the strong correlation of BNP and NT-proBNP with age and renal function, a correlation not observed with copeptin [147].

Copeptin has been demonstrated to play a significant role in both the diagnosis and prognosis of CKD. A recent study involving 149 patients with moderate CKD showed that elevated serum copeptin concentrations are frequently associated with biopsy-proven calcifications of the medium tunic of blood vessels. Furthermore, multiple analyses have demonstrated that copeptin correlates with the degree of vascular calcification regardless of age, gender, or co-affliction with diabetes [148]. Various studies have shown that circulating levels of copeptin, independent of GFR, predict an increased risk of CKD through two mechanisms [142]. Firstly, reduced GFR leads to the plasmatic accumulation of copeptin due to its renal elimination. Secondly, renal dysfunction determines the secretion of AVP (implicitly of copeptin as well) due to the low ability to concentrate urine in order to maintain water homeostasis [142,144].

But, at the same time, there are studies that demonstrate an increase in copeptin levels before a decrease in GFR in some patients, suggesting that there may be another, yet unidentified kidney-damaging mechanism. Recently, a reported relationship between insulin resistance, copeptin, and CKD indicated that peripheral insulin resistance is associated with increased copeptin levels in non-diabetic patients with moderate CKD [142,149]. This hypothesis suggests a potential pathogenic connection between AVP and insulin resistance in CKD. From a prognostic perspective, studies have shown that CKD patients with elevated copeptin levels have a higher risk of experiencing cardiovascular events, with an increased risk becoming more evident with the progression of renal dysfunction [142].

Instead, copeptin determination could be considered an early diagnostic and prognostic tool for patients with CKD. In the healthy population, there is a negative relationship between copeptin and GFR; elevated circulating levels of copeptin may independently predict an increased risk of CKD [142]. The diagnostic role of copeptin in distinguishing nephrogenic diabetes insipidus from that caused by inadequate ADH secretion is well-established. Furthermore, in CKD patients, elevated copeptin levels have been linked to an increased risk of cardiovascular disease and mortality.

### 3.6. Fibroblast Growth Factor 23 (FGF23) and Klotho

Fibroblast growth factor 23 (FGF23) is a protein secreted by osteocytes in bone, and it serves as a potent regulator of vitamin D and phosphate metabolism [150]. Additionally, FGF23 functions as a counter-regulatory hormone for vitamin D by impeding the production of 1,25(OH)2D [93,151]. Klotho acts as a coreceptor for FGF23, and its presence seems essential to induce FGF23-specific signaling pathways in the kidneys, parathyroid glands, and other tissues. When Klotho is enzymatically cleaved and released into the circulation, it becomes a phosphaturic and hypocalciuric hormone independent of FGF 23 [93]. FGF23 suppresses the renal synthesis of 1,25-dihydroxy vitamin D, leading to a decrease in serum phosphate levels. Moreover, it inhibits the secretion of parathyroid hormone (PTH) from the parathyroid glands [152].

**Figure 3:**
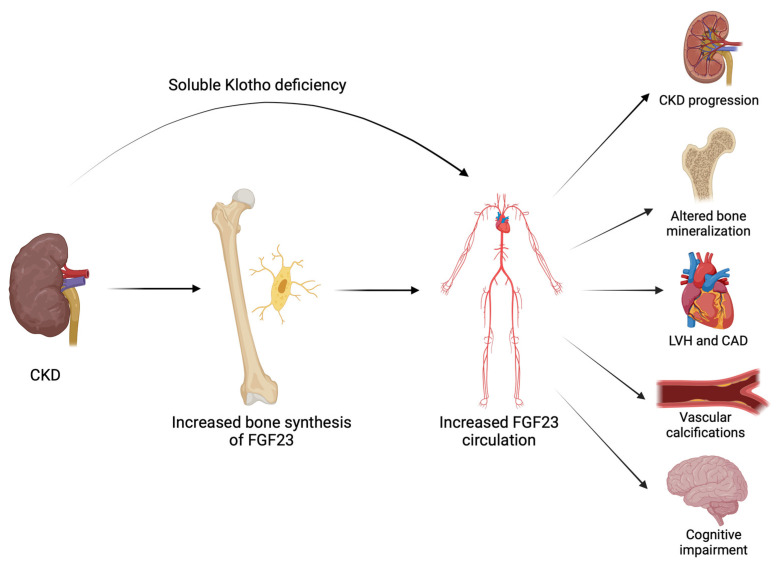
Action mechanism of FGF 23 and Klotho. This image was created with BioRender.com (accessed on 18 November 2023).

Secondary hyperparathyroidism is a specific complication of CKD. The global syndrome involving bone and cardiovascular damage in CKD has been named CKD-mineral bone disorder (CKD-MBD) [93]. It is characterized by vitamin D deficiency, initially leading to hyperphosphatemia and hypocalcemia, followed by hypercalcemia. Among all the parameters of mineral metabolism, FGF23 changes first, serving as the initiator of the CKD-MBD cascade. Elevated levels of FGF23 occur begining in stage 2 of CKD, and the increase in FGF23 aligns with the decrease in GFR. As renal dysfunction progresses, the expression of Klotho decreases. With the reduction in the number of viable nephrons, the excretory capacity of phosphorus is no longer sufficient despite the high level of FGF23. Consequently, renal resistance to FGF23 develops, associated with a dramatic increase in the plasma concentration of FGF23 (see Figure 3) [93,150,153].

In CKD patients, there is an adverse association between elevated plasma FGF23 levels and cardiovascular damage. Studies indicate that FGF23 serves as a predictor for heart failure progression and acute atherosclerotic cardiovascular events. Left-ventricular dysfunction is of particular concern with respect to its correlation with increasing levels of FGF23. Approximately 74% of CKD patients, especially those with ESKD, exhibit forms of pathological LV remodeling. LVH, in this case, predisposes individuals to sudden cardiac death and the development of heart failure with preserved or reduced ejection fractions [150]. Mirza et al. (PIVUS cohort) and Faul et al. demonstrated a positive correlation between elevated serum FGF23 levels and LVH based on imaging documentation. In vivo experiments conducted by Faul et al. revealed that the intramyocardial injection of FGF23 in mice causes left-ventricular hypertrophy independent of Klotho, which is not expressed by cardiac myocytes. However, in mice expressing Klotho receptors, LVH was more modest, suggesting a potential loss of the protective effect of Klotho receptors or their deficiency in CKD patients.

Alternatively, the myocardial effects of FGF23 could be mediated by another pathway, such as the FGFR4 activation of the PLC/calcineurin/NFAT pathway [150,154]. Investigations involving 3070 patients from the CRIC (Chronic Renal Insufficiency Cohort) study demonstrated a direct association between decreased GFR and increased FGF23 and LVH levels. Furthermore, experimental evidence showed that FGF23 phosphorylates phospholipase C gamma (PLCγ) via binding to FGFR isoform 4 on cardiac myocytes independently of Klotho. PLCγ phosphorylation leads to the activation of the calcineurin/NFAT signaling cascade, inducing prohypertrophic gene programs both in vitro and in vivo [148,154,155]. Various mechanisms by which FGF23 contributes to ventricular arrhythmias, impaired calcium signaling, the activation of the renin–angiotensin aldosterone system, and myocardial fibrosis have been elucidated [152].

Multiple studies have demonstrated a correlation between plasma FGF23 levels and vascular damage, particularly in the coronary arteries. In a cross-sectional study involving 177 patients with mild to moderate CKD undergoing coronary angiography, an early increase in FGF23 was found to be an independent predictor of CAD severity [93,156]. Additionally, research indicates that the initial vascular abnormality in patients with mild CKD, accompanied by elevated FGF23 levels, is endothelial dysfunction associated with oxidative stress. In stages 3–4 of CKD, elevated FGF23 levels are independently associated with decreased vasodilation. However, no association of FGF23 with endothelial function was observed in dialysis patients [152]. Clinical studies have highlighted that vascular calcification is an independent predictor of cardiovascular mortality in CKD.

The Klotho coreceptor exerts direct vascular effects by enhancing nitric oxide production in endothelial cells and promoting the generation of reactive oxygen species in vascular smooth muscle cells. Consequently, Klotho deficiency has been linked to heightened oxidative stress and inflammation, particularly in patients with advanced forms of CKD [93,157]. In a large observational study involving 804 patients, those with the lowest Klotho levels had a 78% higher risk of death than those with elevated levels [140]. In a cross-sectional study of 956 individuals, Klotho gene polymorphisms were associated with occult CAD, independent of CAD risk factors. Recently, a correlation was reported between the severity of CAD and reduced Klotho gene expression in the aorta [93].

At present, a dependable test for measuring Klotho is not available, and despite the earlier findings, the evidence supporting the effectiveness of FGF-23 and Klotho as risk markers is inconclusive. Therefore, they cannot be recommended for routine clinical use. Furthermore, the re-expression of endogenous Klotho synthesis in the kidneys or supplementation with exogenous Klotho have been shown to attenuate vascular calcification in CKD. This suggests that CKD-MBD is not solely due to excess FGF23 but that an imbalance between FGF23 and its cofactor Klotho might be involved [152]. A study involving 227 patients diagnosed with CKD demonstrated a significant association between plasma FGF23 concentration and GFR. After approximately 4 years of follow-up, patients with elevated FGF23 levels showed a faster progression of CKD and a worse prognosis [152]. In another large study (ARIC—Atherosclerosis Risk in Communities), which included 13,000 participants, increased baseline levels of FGF23 correlated with a higher risk of ESKD after approximately 21 years of follow-up, even after adjusting for risk factors [152,158]. Moreover, multiple studies have shown that the chronic inflammation characteristic of CKD patients stimulates the synthesis of FGF23, creating a vicious circle by inducing inflammatory markers [152].

Therefore, both FGF23 and its coreceptor Klotho are crucial biomarkers for CKD progression and assessing the severity of cardiovascular damage secondary to CKD-MB. They also provide important prognostic information on both pathologies [152].

### 3.7. Plasma Growth Differentiation Factor 15 (GDF-15)

Growth differentiation factor-15 (GDF-15) is a protein belonging to the GDF subfamily of the transforming growth factor-β (TGF-β) superfamily [159,160]. It is naturally present in cells of the cardiovascular, immune, and renal systems. In typical physiological conditions, the placenta is the main organ that secretes a substantial amount of GDF-15, especially during the third trimester [159,161]. Overexpression of GDF-15 occurs in response to pathophysiological processes such as inflammation, ischemia, and mechanical or oxidative stress. This protein is commonly expressed in cardiovascular, pulmonary, renal, and neoplastic diseases [162].

Numerous studies have indicated that elevated levels of GDF-15 predict all-cause mortality in both chronic and acute heart failure. GDF-15 can also forecast the rate of hospitalizations and deterioration of cardiovascular function by correlating with echocardiographic indices [163]. In a large community-based study, a change of more than 40% in the concentration of GDF-15 was associated with a fourfold increased risk of mortality [163,164]. GDF-15 is expressed in various tissues, and its expression is regulated by the p53 enzymatic system. Most evidence suggests the involvement of GDF-15 in cardiovascular and cancer mortality, where the key processes include oxidative stress, inflammation, and repair. Considering that p53 is a mediator in cardiovascular pathology and cancer, it is hypothesized that increased levels of this biomarker could indicate the need for repair even before the appearance of specific organ damage, potentially facilitating early intervention [163].

GDF-15 has also proven to be a strong and independent predictor of mortality and progression in chronic coronary syndromes as well as in acute coronary syndromes. The plasma concentration of GDF-15 increases within a few hours of the onset of a myocardial infarction and remains elevated for several days. In patients with N-STEMI, GDF-15 enhances the predictive value of GRACE (Global Registry of Acute Coronary Events) scores even more than NT-proBNP, and it is useful in risk stratification concerning the choice of invasive treatment [92,165]. Additionally, multiple studies have demonstrated that increased levels of GDF-15 are associated with the presence of stable CAD, with higher concentrations found in patients with multivessel coronary disease and those with a history of myocardial infarction. Recently, Gohar et al. revealed that elevated GDF-15 values predict major cardiovascular events in women with carotid atherosclerosis [162].

The role of GDF-15 as a biomarker in CKD is still being explored. Hyperexpression of this molecule has been observed in the kidneys of patients diagnosed with CKD. Multiple studies have indicated a positive correlation between plasma GDF-15 levels and the incidence of CKD. This correlation is partly attributed to the increased level of GDF-15 in the kidneys, serving as a protective response against early-stage kidney damage [159]. Notably, data from the Framingham Offspring study, involving 2614 participants observed over a follow-up of approximately 9 years, revealed a significant association between high levels of GDF-15 and the incidence of CKD as well as a rapid decline in GFR [93]. Another study identified increased GDF-15 levels as an independent predictor of cardiovascular mortality and worsening renal function in patients with diabetic nephropathy [93].

Therefore, GDF-15 is recognized as a biomarker with strong predictive value for prognosis and all-cause mortality for patients with ischemic heart disease or heart failure. As a renal biomarker, GDF-15 shows promise in identifying individuals at risk of developing CKD and serving as a prognostic indicator for the progression and mortality of CKD patients.

## 4. Future Perspectives

The inclusion of cardiac and renal biomarkers in multimarker strategies is a promising approach to managing cardio-renal interactions, as it leads to increased accuracy of individual markers for diagnostic, prognostic, and therapeutic purposes [166]. However, the proper selection, application, and combination of biomarkers represent a complex yet promising process that, in the future, can be integrated into clinical practice. Promising biomarkers include plasma thiobarbituric acid-reactive substances, 8-epi-isoprostanes, soluble thrombomodulin, and Angpt2, which, according to studies, have been found to be elevated in patients with cardiovascular and renal impairment [166,167]. Furthermore, mid-regional proadrenomedullin (MR-proADM) and liver-type fatty-acid-binding protein (L-FABP) have been able to predict decline in renal function in patients with associated cardiovascular conditions, especially heart failure [166]. Urinary cofilin-1 is a newly discovered biomarker with a role in predicting acute renal dysfunction in patients with chronic cardiovascular conditions [166,168].

New therapeutic strategies have considered the activation of the erythropoietin receptor at the cardiac level. In patients with coexisting chronic kidney disease (CKD), anemia, and heart failure, it induced antiapoptotic, antifibrotic, and anti-inflammatory effects, resulting in an improvement of cardiac structure and function [166,169]. A promising strategy in managing the cardiorenal syndrome is the use of SGLT2 inhibitors. According to studies, in addition to their pleiotropic effects on the cardiovascular system, these medications slow down the progression of renal dysfunction, regardless of the presence of type 2 diabetes mellitus. 

Randomized controlled trials have shown that canakinumab treatment could lead to a reduction in cardiovascular events, especially in patients who experienced an acute myocardial infarction and had CKD. However, the studies conducted so far have included patients with mild to moderate CKD [29,45]. Additionally, research on the inhibition of the proinflammatory cytokine IL-1β in patients with concomitant chronic kidney disease and cardiovascular impairment is promising [29,170]. Moreover, patients with CKD are subjected to affliction with significant nephrotoxic and cardiotoxic statuses, secondary to the accumulation of uremic toxins. For this reason, identifying a strategy for preventing the accumulation of uremic toxins is necessary and is currently under investigation as a therapeutic target for the cardio-renal syndrome [29].

Notably, an important strategy would involve conducting studies on patients with chronic kidney disease, classified according to stage, including those undergoing dialysis therapy, to assess the effectiveness and safety of various therapeutic and diagnostic methods.

## 5. Conclusions

Chronic kidney disease associated with ischemic heart disease remains one of the most challenging pathophysiological interactions. Due to the overlap of the mechanisms presented earlier, differentiating primary and secondary disease can be complicated. However, this distinction can play a crucial role in the development of new treatments to improve both cardiac and renal functions.

In this review, we focused on understanding the pathological mechanisms underlying the interaction between chronic kidney disease and ischemic heart disease, as well as the analysis of common biomarkers for both pathologies, such as cardiac troponins, natriuretic peptides, copeptin, galectin-3, FGF-23 and Klotho, sST2, and GDF-15, which have proven their diagnostic and prognostic roles. Developing a multi-marker strategy for a more precise quantification of risk for these patients is important.

Moreover, for patients with chronic kidney disease, especially those in advanced stages, diagnosis and treatment remain a true challenge, even with the progress made so far. The challenge in relation to these patients arises from the fact that they are often excluded from studies and clinical trials due to complications that may arise from severely impaired renal function. 

## Data Availability

Data sharing is not applicable.
